# War Savings Associations

**Published:** 1916-10-07

**Authors:** M. C. Sparshott

**Affiliations:** Lady Superintendent of Nurses. Royal Infirmary, Manchester.


### THE EDITOR'S LETTER-BOX.

### War Savings Associations.

*To the Editor of* THE HOSPITAL.

SIR,—Friendly rivalry is good in all things, even in "War Savings." I see from THE HOSPITAL (September 30) that a London hospital has done well. We have done even better.

We started on September 30, and have obtained, from nurses and female servants alone, £120.

Wishing luck to other hospitals, I remain yours faithfully,

M. C. SPARSHOTT,
Lady Superintendent of Nurses.

Royal Infirmary, Manchester.
October 3, 1916.

